# Do Thyroid Diseases during Pregnancy and Lactation Affect the Nutritional Composition of Human Milk?

**DOI:** 10.1055/s-0040-1718449

**Published:** 2020-11-30

**Authors:** Fernanda de Oliveira Lopes, Fernanda Valente Mendes Soares, Danielle Aparecida da Silva, Maria Elisabeth Lopes Moreira

**Affiliations:** 1 Instituto Nacional da Saúde da Mulher, da Criança e do Adolescente Fernandes Figueira, Fiocruz, Rio de Janeiro, RJ, Brazil

**Keywords:** human milk, macronutrients, composition, thyroid gland diseases, hypothyroidism, leite humano, macronutrientes, composição, doenças da glândula tireoide, hipotireoidismo

## Abstract

**Objective** To identify whether the effects of thyroid disease during pregnancy and lactation affect the nutritional composition of human milk.

**Methods** Systematic review of the scientific literature using the Medical Literature Analysis and Retrieval System Online/MedLine databases to evaluate the association of thyroid diseases during pregnancy and lactation with the nutritional composition of human milk. There was no delimitation by period or by language, and the searches were completed in March 2019. The following descriptors were applied: *human milk* AND *thyroid* AND *composition* , using the preferred reporting items for systematic reviews and meta-analyses (PRISMA) protocol for data search, selection, and extraction. The flowchart proposed for bibliographic search resulted in 12 articles and, of these, four were selected.

**Results** The articles elected for this review were published between 1976 and 2018. Two studies found significant differences in the nutritional composition of mothers' milk with hypothyroidism or overweight compared with the milk of those without hypothyroidism. Studies have shown that the presence of the disease led to changes in the nutritional composition of human milk, especially a higher concentration of human milk fat.

**Conclusion** It is extremely important that these women have continuous nutritional follow-up to minimize the impact of these morbidities on the nutritional composition of human milk.

## Resumo

**Objetivo** Identificar se os efeitos da doença da tireoide durante a gestação e lactação afetam a composição nutricional do leite humano.

**Métodos** Revisão sistemática da literatura científica por meio das bases de dados *Medical Literature Analysis and Retrieval System Online/MedLine* a fim de avaliar a associação das doenças da tireoide na gestação e na lactação com a composição nutricional do leite humano. Não houve delimitação por período nem por idioma, e as buscas foram finalizadas em março de 2019. Foram aplicados os seguintes descritores: *human milk* AND *thyroid* AND *composition* , utilizando protocolo *preferred reporting items for systematic reviews and meta-analyses* (PRISMA) para a busca, seleção e extração de dados. De acordo com o fluxograma proposto, a busca bibliográfica resultou em 12 artigos e, destes, quatro foram selecionados.

**Resultados** Os artigos elegidos para a presente revisão foram publicados entre 1976 e 2018. Dois estudos verificaram diferenças significativas na composição nutricional do leite de mães com hipotireoidismo ou excesso de peso em comparação ao grupo controle sem hipotireoidismo. Os estudos demonstraram que a presença da doença levava a modificações na composição nutricional do leite humano, principalmente em relação à maior concentração da gordura.

**Conclusão** É de extrema importância que essas mulheres tenham acompanhamento nutricional contínuo a fim de minimizar o impacto dessas morbidades sobre a composição nutricional do leite humano.

## Introduction

It is known that thyroid hormones act on the growth and development of children and adolescents, having a fundamental role in the brain development of the fetus and the newborn, in body weight. 1 At the cellular level, they participate in the positive regulation of carbohydrate, in lipid catabolism, and in the stimulation of protein synthesis in a wide variety of cells, and it acts in most tissues. 2 Over the years, studies have observed that among the diseases of the thyroid gland, hypothyroidism is the most common endocrine alteration, mainly in reproductive age. 3 4 5 6 7 8 In Brazil, according to data from the Ministry of Health, hypothyroidism has a prevalence of 2% in the general population, being 8 times more frequent in women, with primary hypothyroidism being the main form of occurrence in almost 95% of the cases. 7 8 Hypothyroidism may also be related to the increase in obesity, since it is characterized by excess body fat resulting from a positive energy balance capable of inducing the development of this comorbidity. 9 10 11 12

Breastfeeding is recognized nationally and internationally as the safest and most effective method of feeding babies. 13 Human milk (HM) consists of 87% water, 1% protein, 4% lipids, and 7% of carbohydrates (including 1–2.4% of oligosaccharides). 14 15 It is noteworthy that human milk has its nutritional composition modified in its different phases, which are: colostrum, transition milk, and mature milk. Colostrum is a milk secretion produced from the last trimester of pregnancy and lasts until the 7 ^th^ day postpartum, being a more viscous liquid with a high concentration of proteins and less fat and energy. Transitional milk, on the other hand, is produced between the 7th and 14th days postpartum, with a decrease in protein concentration, and an increase in lactose, fat, and energy levels. And we have mature milk from the 15th day postpartum, and its characteristic is a waterier secretion. Understanding the composition of HM provides an important tool for managing infant feeding, particularly for preterm babies. 16

It is important to say that gestational hypothyroidism is one of the most common thyroid diseases among pregnant women and that human milk is a very important factor for child health. It is worth noting that harmful effects for both women and newborns cannot be ignored. Some studies show that gestational hypothyroidism is associated with some diseases, such as severe preeclampsia, gestational diabetes, placental detachment, higher incidence of premature births, increased fetal mortality, slow weight gain and impaired cognitive development. 17 18 19 20 Human milk provides an important link between mothers and their babies, and the effects of hypothyroidism on lactation are gaining increasing attention.

Studies already performed reported that hypothyroidism directly or indirectly regulates transcription in breast cells, regulating the levels of circulating hormones, such as corticosterone, prolactin, and progesterone, which can cause changes in the quality and quantity of human milk synthesis. 21 This can be attributed to the fact that prolactin promotes the synthesis of milk proteins, such as β-casein and α-lactalbumin, and adequate concentrations of thyroid hormones are essential for milk production in response to prolactin. 22 Another study showed that the level of thyroxine in mothers' plasma is not only positively correlated with the amount of milk production, but also affects milk protein synthesis. 23 However, a more comprehensive analysis of macronutrients in human milk from mothers with thyroid disease still needs to be further investigated.

Although human milk has already been the subject of numerous researches, some questions still need to be clarified, among which: the relationship of maternal thyroid disease with the constituents of the nutritional composition of human milk. Given this, the present study aims, from a systematic review, to verify the impacts of diseases of the thyroid gland on the nutritional composition of human milk.

## Methods

A systematic review of the available scientific literature was performed, which consisted of a retrospective research of articles to assess the association of thyroid diseases in pregnancy and lactation with the nutritional composition of HM.

The following bibliographic databases were used: Medical Literature Analysis and Retrieval System Online / MedLine through PubMed, Biblioteca Virtual da Saúde (Virtual Health Library [VHL]) and Latin American and Caribbean Literature in Health Sciences / Lilacs. It is worth mentioning that the search for the articles was conducted independently by two researchers, and it started in January and ended in March 2019, with an update of the search in November 2019 to find out if there would be new studies to compose the review. For the selected studies, there was no delimitation by publication period. The following keywords were used in the search strategy: human milk, thyroid and composition.

The bibliographic search was performed according to the established strategy and resulted in 118 articles. A total of 101 articles were found in the VHL database, but after reading the title, only 6 were selected; 12 were found in the PubMed database, of which only 6 were selected; and five articles were found in the Lilacs database, none of them being selected. With that, 12 articles were selected for full summary reading, 5 of which were excluded after reading as they did not have a theme for the association of thyroid disease and HM composition. After this reading, we only selected seven manuscripts that were listed for reading in full, and then only three articles were selected that addressed the theme of the nutritional composition of human milk in mothers with thyroid problems. The others were excluded for the following reasons: studies replicated in databases, different thematic association between thyroid disease and HM, and literature review. At this point, an additional search was made based on the bibliographic references of the articles read in full, to increase the sensitivity and to select articles not captured in the electronic search; one more article was selected then, resulting in a total of four studies to compose the present review. A checklist with 27 items and a 4-step flowchart, recommended by the preferred reporting items for systematic reviews and meta-analyses (PRISMA), 24 were used to help authors improve the reporting of systematic reviews.

**Fig. 1:**
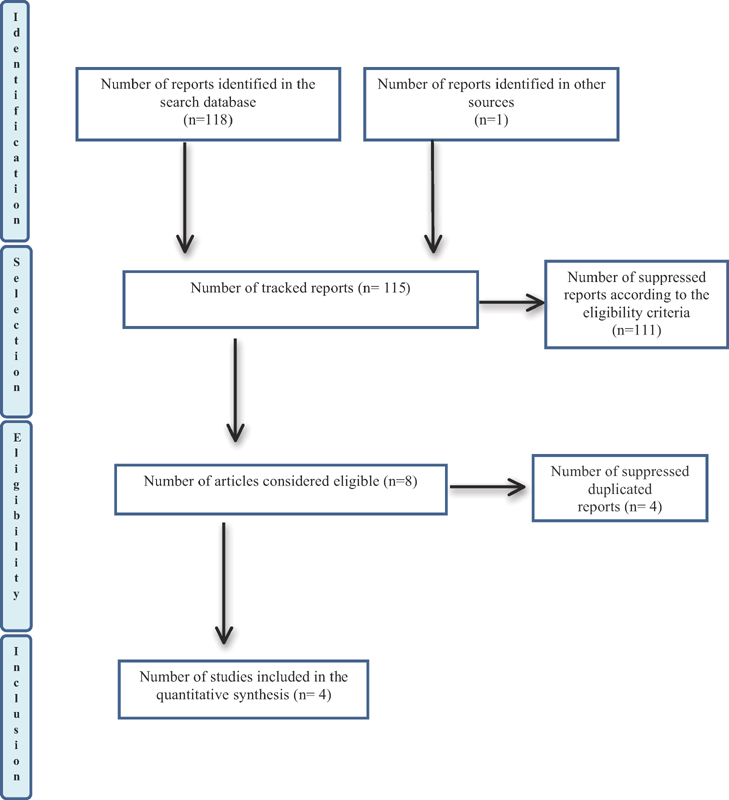
Flow of the selection process for selected articles – preferred reporting items for systematic reviews and meta-analyses (Prisma).

In this way, a summary of each step made in the selection process of the articles that made up this systematic review is shown in the flowchart ( Fig. 1 ).

The following inclusion criteria were considered: original articles in which the nutritional composition of HM in mothers with thyroid problems was considered as an outcome; articles written in Portuguese, English, or Spanish.

The selected articles were compared in relation to the following items: year of publication, place (country/city) of the study, sample size, average age of women, type of design, period when the milk was evaluated, method of analysis of the HM, confounding factors controlled in the analysis, and main observed results.

## Results

Through the search presented above, 4 articles published between 1976 and 2018 were selected that contemplated the nutritional composition of HM in mothers with thyroid problems. Most studies had a small number of participants, ranging from 12 to 30 lactating women. The studies were performed in North America (United States), Oulu (Finland), Beijing - Beijing (China) and South America (Chile). The population studied was aged between 16 and 35 years, and 1 article did not inform the age range of the participants.

Several techniques were used to analyze the composition of HM. Among those used in one of the studies to analyze the fatty acid content was liquid gas chromatography. The analysis of whey protein was made using high resolution mass spectrometry, and the final concentration of whey protein was measured by the bicinconinic acid (BCA) method. Regarding the analyzed human milk phases, it was observed that some studies examined colostrum milk, and others studied both colostrum and mature milk. The number of analyses of milk varied from 1 to 2 times. Only one article cited the exclusion criteria used ( Chart 1 ).

**Chart 1:** Exclusion criteria, method used to evaluate the composition of human milk, and milk phase analyzed in selected studies on maternal thyroid disease and its influence on the nutritional composition of human milk

Authors	Exclusion criteria	Method used to assess the composition of human milk	Milk stage analyzed
Tyson et al. 25	Uninformed	Infrared milk analyzer (IRMA)	Colostrum and mature
Kivinen et al. 26	Uninformed	Gas-liquid chromatography	Colostrum
Motil et al. 27	Uninformed	Kjeldahl method e adiabatic bomb calorimetry	Colostrum
Chen et al. 28	Maternal diseases, fever, mastitis, or diabetes	High resolution mass spectrometry and liquid chromatography mass.	Colostrum

As for the design employed in the studies, all were prospective cohort; one study followed the participants for 1 year, with the follow-up after discharge being performed at the participant's home. Another study followed its participants for 16 weeks postpartum, and this was followed up by nurses-midwives; one accompanied the participants during the postpartum hospitalization period; and the other study monitored the participants from the moment of diagnosis until ∼ 48 hours after birth. Regarding the results found by the selected articles, it was observed that all studies evaluated the nutritional composition of milk, two evaluated data regarding milk production, and only one evaluated data regarding baby growth and maternal weight gain ( Chart 2 ).

**Chart 2:** Type of study, confounding factors, and main results found in selected studies on maternal thyroid disease and its influence on the nutritional composition of human milk

Authors	Year of publication	Type of studyo	Confounding factors	Results found
Tyson et al. 25	1976	Cohort	Obesity	- TRH administration was not associated with changes in the percentage of protein and milk fat between groups in the postpartum period, but after the 1st and 12th postpartum weeks a significant decline in the average protein concentration was observed. - There was no difference in the weight of infants and lactating women between groups. - Lactating mothers who received TRH showed an increase in milk production
Kivinen et al. 26	1979	Cohort	Age and parity	It was found that the fatty acid content of milk samples from women treated with TRH did not differ from that of normal lactating women.
Motil et al. 27	1994	Cohort	Uninformed	- It has been found that insulin modulates the concentration of nutrients between the anabolic and catabolic aspects of mothers' protein metabolism, while thyroid hormones and cortisol modulate the partition of nutrients for milk production and protein synthesis. - It has been observed that some non-essential amino acids become limiting during lactation because of their unique contributions to milk protein synthesis.
Chen et al. 28	2018	Cohort	Uninformed	The results suggest that hypothyroidism can alter the serum protein of human colostrum at the composition level, decreasing the levels of metabolic proteins and proteins of cellular structure, while increasing the levels of proteins related to the immune system, which can compromise or reflect in the health of mothers and babies.

Abbreviation: TRH, thyrotropin-releasing hormone.

Only one study reported that the women who participated in the research were diagnosed with gestational hypothyroidism through examinations in the first trimester of pregnancy, and, thus, initiated replacement treatment (thyroxine) during pregnancy. 28 Regarding withdrawal of milk samples to be analyzed, 3 studies reported that they performed the collection after 24 hours postpartum, 25 27 28 and 1 study performed its collection as early as 2 hours postpartum. 26 As for how this milk was withdrawn, a study reported that it was performed with a breast pump and manually on each breast, 28 one used the milk removal technique only with a breast pump and this removal occurred at the same time that the newborn was breastfeeding on the other breast. 27 Another study reported that the collection only occurred manually, always starting on the right breast and then moving to the left breast, withdrawing ± 10 ml of previous milk. 25 Only one study did not report how they removed the milk for analysis. 26

Regarding the composition of milk, Kivinen et al., 26 in their study, did not observe statistically significant differences regarding fatty acid. Motil et al., 27 in their study, showed that there were positive associations between the hormones thyroxine and triiodothyroxine and the amount of milk produced as well as in the composition of milk at 1, 5, and 12 months after delivery. It is worth mentioning that this was the only study that did a control in maternal nutrition, through a constant and controlled diet of proteins and energy content.

Tyson et al. 25 observed in their study that when assessing the weekly weight of infants in the groups of treated and untreated mothers in the first week, there was weight gain in the infants; however, when evaluating these same groups at 12 and 16 weeks, it was found that there was no difference in infant weight gain from mothers who received oral tripeptide.

Tyson et al. 25 evaluated that the administration of thyrotropin-releasing hormone (TRH) was not associated with changes in the composition of HMin terms of the percentage of protein and fat at birth when compared with the control group that did not receive TRH; however, when observed between the 1st and 12th weeks postpartum, there was a gradual and significant decline in mean protein concentration. Chen et al. 28 showed that hypothyroidism can alter the composition of whey protein in the period of colostrum. These authors also evaluated that hypothyroidism increases the levels of protein related to the immune system, which can compromise or reflect on the health of mothers and babies.

## Discussion

At this moment, the main results found in the articles selected for this systematic review will be argued and highlighted, focusing on the possible effect that the thyroid disease (hypothyroidism or hyperthyroidism) has on the nutritional composition of HM. As the studies that participated in this review when analyzing their data used very different methods from each other to evaluate the composition of HM, the discussion of each one occurred separately, each being confronted with the available literature.

As reported by Tyson et al., 25 there was no difference in the percentage of protein, fat, and lactose in the nutritional composition of HM until the 1st week postpartum, but from the 1st to the 12th weeks postpartum, a gradual and significant decline in the average concentration of milk protein was observed. Supporting the study above, this decline was also observed in a study by Lönnerdal (2003), 29 in which he states that the protein content of HM decreases rapidly during the first month of lactation and then continues to decrease as well, only more slowly, after the first month. Another study that also corroborates the statement above was that of Bauer and Gerss (2011), 30 in which they found that protein levels decrease in HM in the first 4 to 6 weeks or more, regardless of the time of delivery.

The observation reported by Kivinen et al., 26 in their study, that they found no change in the composition of breast milk fat, in which the main fatty acids evaluated were lauric acid (C12: 0), myristic acid (C 14: 0), palmitic (C16: 0), palmitoleic acid (C 16: 1), stearic acid (C 18: 0), oleic acid (C 18: 1), linoleic acid (C18: 2), and linolenic acid (C 18: 3) it is very important for the growth and development of the newborn, since ∼ 50% of the total energy intake is acquired from milk lipids during the 1st month after birth, and body fat is responsible for 35% of the weight gain of a child during the first 6 months, as several studies show. 31 32 33

Now, another study that made up this review showed that there was a change in the composition of whey protein in the colostrum of women with gestational hypothyroidism, in whom a decrease in the levels of metabolic proteins was observed. 28 This change can be justified, because the processes and pathways that mediate the metabolism of carbohydrates, lipids, and proteins are all affected by thyroid hormones in almost all tissues, 34 and since these hormones are galactopoietic and help to establish the metabolic priority of the mammary gland during lactation as well as in the gestation period, the body mobilizes these hormones to prepare the breast for lactation, thus worsening the proper function of the mammary glands. 22

The statement by Motil et al. 27 that thyroid hormones modulate the partition of nutrients for milk production, composition and protein synthesis of mature milk, as well as some non-essential amino acids becoming limiting during lactation because of their unique contributions to milk protein synthesis, and significant positive associations in the amount of milk production. A study that contrasts with the one reported above is the study by Hart et al. (1978) 35 with cattle whose plasma concentrations of thyroxine were inversely related to milk production. This being the only article in the present review that controlled the maternal diet for protein and energy intake, this variation makes one wonder what may be altering this process, since protein and carbohydrate are macronutrients that do not change with the maternal diet. 16

It can be observed in the present systematic review, that protein was the macronutrient that showed the greatest changes in its composition, being a very important component of HM, as it acts on the growth and development of newborn's muscle mass.

The divergent results found in the studies selected for this article can be relatively explained by the methodological differences presented in each study, such as: method used in the evaluation of HM, nutritional components evaluated, lactation stage (colostrum, transition and mature), and sample size. The limitations present in this article were: the small number of references on the topic and the lack of more current studies, leaving a very large gap in time, since in the search only one study was from the year 2018, two from the 70s and one of the 90s. Considering the above mentioned limitations, it is important that new studies are performed to evaluate the association of diseases of the thyroid gland on the nutritional composition of HM.

## Conclusion

Although the studies have been disparate in several methodological aspects, it is extremely important that women have continuous nutritional and medical monitoring in the prenatal and postpartum periods to have an effective control of thyroid disease and thus minimize changes that it can cause on the nutritional composition of HM. New studies related to this theme are needed to evaluate if there are changes to other components of HM, aiming to improve even more the approach with the mother-baby binomial. It is worth mentioning that although thyroid diseases cause nutritional changes in HM, all studies and the World Health Organization are in full agreement that breastfeeding should be encouraged exclusively until the age of 6 months and complemented by 2 years or more. 15 16
